# Qualitative Analysis of Responses in Estimating Older Adults Cognitive Functioning in Spontaneous Speech: Comparison of Questions Asked by AI Agents and Humans

**DOI:** 10.3390/healthcare12212112

**Published:** 2024-10-23

**Authors:** Toshiharu Igarashi, Katsuya Iijima, Kunio Nitta, Yu Chen

**Affiliations:** 1 Simulation of Complex Systems Lab, Department of Human and Engineered Environmental Studies, Graduate School of Frontier Sciences, The University of Tokyo, Chiba 277-0882, Japan; 2 AI-UX Design Research Institution, Advanced Institute of Industrial Technology, 10-40 Higashi-Oi 1-Chome, Shinagawa, Tokyo 140-0011, Japan; 3 Institute of Gerontology (IOG), The University of Tokyo, Tokyo 113-8656, Japan; 4 Institute for Future Initiatives (IFI), The University of Tokyo, Tokyo 113-0033, Japan; 5 Tsukushikai Medical Corporation, Tokyo 186-0005, Japan

**Keywords:** AI agents, cognitive function estimation, Alzheimer’s disease, natural language processing

## Abstract

Background/Objectives: Artificial Intelligence (AI) technology is gaining attention for its potential in cognitive function assessment and intervention. AI robots and agents can offer continuous dialogue with the elderly, helping to prevent social isolation and support cognitive health. Speech-based evaluation methods are promising as they reduce the burden on elderly participants. AI agents could replace human questioners, offering efficient and consistent assessments. However, existing research lacks sufficient comparisons of elderly speech content when interacting with AI versus human partners, and detailed analyses of factors like cognitive function levels and dialogue partner effects on speech elements such as proper nouns and fillers. Methods: This study investigates how elderly individuals’ cognitive functions influence their communication patterns with both human and AI conversational partners. A total of 34 older people (12 men and 22 women) living in the community were selected from a silver human resource centre and day service centre in Tokyo. Cognitive function was assessed using the Mini-Mental State Examination (MMSE), and participants engaged in semi-structured daily conversations with both human and AI partners. Results: The study examined the frequency of fillers, proper nouns, and “listen back” in conversations with AI and humans. Results showed that participants used more fillers in human conversations, especially those with lower cognitive function. In contrast, proper nouns were used more in AI conversations, particularly by those with higher cognitive function. Participants also asked for explanations more often in AI conversations, especially those with lower cognitive function. These findings highlight differences in conversation patterns based on cognitive function and the conversation partner being either AI or human. Conclusions: These results suggest that there are differences in conversation patterns depending on the cognitive function of the participants and whether the conversation partner is a human or an AI. This study aims to provide new insights into the effective use of AI agents in dialogue with the elderly, contributing to the improvement of elderly welfare.

## 1. Introduction

Japan has entered a super-ageing society, and the welfare and maintenance of cognitive functions of the elderly are important issues. In Japan, as of 2020, the elderly population aged 65 and over accounted for about 28% of the total population, and it is predicted to exceed 30% by 2030 [1]. Cognitive decline leads to a decrease in the quality of life and an increase in the burden of caregiving, requiring early detection and appropriate intervention. Recent studies have shown that appropriate social and cognitive stimulation contributes to the maintenance and improvement of cognitive function [2].

Many cognitive function tests for diagnosing cognitive impairments have issues such as testing time, invasiveness, psychological burden on patients, and costs associated with the necessary equipment and facilities. CT and MRI scans can only be performed in large hospitals with such facilities, and there are issues with the testing time, cost burden, and invasiveness for patients. Neuropsychological tests can be performed in a relatively short periods of time, but they require clinical psychologists with specialized knowledge, and the results can be affected by the patient’s condition. According to Miller et al.’s research, these tests can cause stress and anxiety in test-takers. For example, the results of psychological tests are often used for important decision-making, so test-takers may feel pressured [3]. Furthermore, the American Psychological Association (APA) addresses the burden on test-takers during psychological testing. The APA guidelines emphasize the potential adverse effects of conducting psychological tests, assessments, and measurements, particularly in high-pressure or uncertain situations that could significantly impact an individual’s future [4].

Recently, the potential for cognitive function assessment and intervention using AI technology has attracted attention. Robots and agents using AI are expected not only to provide continuous opportunities for dialogue with the elderly but also to prevent their social isolation and contribute to the maintenance and improvement of cognitive functions. These technologies could become important tools for improving quality of life for the elderly. For example, communication support systems using AI can potentially reduce social isolation among the elderly. These systems utilize voice recognition and natural language processing technology to facilitate conversations with the elderly [5]. Additionally, communication via robots and virtual agents equipped with AI can provide opportunities for daily dialogue and information exchange for the elderly [6]. Evaluation methods based on natural speech are especially promising because they can be conducted without burdening the elderly. AI agents have the potential to replace human questioners and are expected to serve as an efficient and consistent means of cognitive function assessment.

This study investigated the impact of dialogue with AI agents and human questioners on the speech content of the elderly. Specifically, the study targeted elderly individuals from a silver talent centre and a day service centre, comparing the number of fillers, proper nouns, and instances of listening back in conversations with humans and AI. The participants included 12 males and 22 females, totaling 34 individuals. The recordings of each conversation were analyzed, and quantitative data were collected. This study aims to provide new insights into the effective use of AI agents in dialogue with the elderly, contributing to the improvement of elderly welfare.

## 2. Related Works

### 2.1. Dialogue Experiments with Communication Robots and AI Agents

Recent studies have investigated the impact of dialogues with communication robots and AI agents on elderly social participation and cognitive functions. For example, Tsujikawa et al. (2023) designed interactions between humans and robots aimed at providing psychological support for elderly individuals living alone, analyzing conversation flows composed of daily topics, physical topics, and psychological topics. As a result, 87% of the participants found the robot useful in daily life, and 87% wished to continue using it [7]. Igarashi et al. (2022) pointed out that appropriate technology is necessary for realizing a society where elderly individuals can lead independent lives for as long as possible [8]. They developed a self-disclosure function to promote continuous interaction with robots and conducted dialogue experiments, finding that the self-disclosure function significantly improved the quantity and quality of verbal interactions with the elderly. Additionally, there are studies comparing physical robots and virtual robots to investigate how elderly individuals engage in dialogue, finding that physical robots had a higher engagement level in dialogues with the elderly [9]. Other studies used robots designed to avoid silence by having the robots continue conversations when users did not respond [10].

Conversational virtual agents are used as avatars with human or other visual appearances [11]. These agents use machine learning and natural language processing to interact with humans on mobile-, web-, and audio-based platforms, and are utilized for cognitive function diagnosis and dialogue with the elderly. In the UK, a study using prerecorded questions for dialogue between a virtual agent and individuals with mild cognitive impairment and healthy controls revealed that the mild cognitive impairment group had shorter speaking times compared to the healthy group, suggesting that voice analysis can contribute to the diagnosis of cognitive impairments [12]. Furthermore, a study developed a voice-controlled smart home system for the elderly, confirming that interactive agents providing natural interactions are useful for daily life support [13]. Rodríguez et al. suggested that agents can reduce feelings of social isolation and provide cognitive support [14]. Also, Schöbel, Sofia, et al. analyzed barriers and promoting factors for the use of voice assistants by older adults, showing that improved usability increases their adoption of such agents [15].

However, these studies do not sufficiently compare the impact on the speech content of the elderly when the dialogue partner is an AI agent or a human. Additionally, specific analyses of the data obtained from responses, such as the influence of cognitive function levels and dialogue partners on the frequency of elements like proper nouns and fillers, are not sufficiently found in previous research. Furthermore, there are still challenges in implementing these technologies, such as user acceptance, privacy issues, and the need for customized solutions for different elderly groups [16]. Elderly acceptance of technology is influenced by several factors, including perceived benefits, ease of use, social support, and individual characteristics [17,18]. This study may provide one suggestion in examining the impact of AI agent technology on the quality of life of the elderly. 

### 2.2. Fillers in Conversation

In conversation, elements like “um” or “uh” that do not have inherent meaning are called fillers and are used to maintain the flow of conversation or fill the gaps between utterances [19,20]. Fillers are said to have various functions, and previous research has discussed their role in maintaining speaking rights [21] and softening interactions [22]. They are also categorized into three functions: expressing the speaker’s information processing ability, contributing to text structure, and relating to interpersonal relationships [20]. Additionally, studies have identified factors such as age and gender that affect the frequency of fillers [23]. Furthermore, research has examined the impact of filler usage, indicating that the frequency of filler uses influences trustworthiness and understanding by the listener [24].

Fillers in conversation serve multiple functions and are influenced by the speaker and the context. For example, when the speaker is a student, fillers can help improve their speaking abilities, boosting confidence and fluency [25]. Additionally, in human–robot interactions, fillers can speed up turn-taking [26]. The impact of fillers also varies for non-native speakers, where repetition and self-repair may not serve as indicators of fluency but rather function as a means of managing conversation [27]. These findings suggest that the role and perception of fillers in conversation are context-dependent and influenced by factors such as the speaker’s proficiency, the medium of communication, and the setting of the conversation. However, it can be said that research focusing on elderly individuals in Japan remains insufficient.

### 2.3. Impact of Cognitive Decline on Conversation

Cognitive decline with ageing affects various cognitive functions, including language abilities. Studies suggest that language impairments may serve as early indicators of mild cognitive impairment (MCI) and dementia [28]. According to previous research, cognitive decline affects communication. In verbal fluency tests, as the severity of dementia increases, both common and proper noun categories show a progressive decline, suggesting impairment in language processing [29]. In Alzheimer’s disease (AD), an increase in empty pauses during speech, especially at the beginning of speech, has been observed as an indicator of cognitive decline [30]. Furthermore, studies focusing on fillers in the speech of Alzheimer’s disease patients have been conducted, showing that the frequency of using ‘um’ is significantly lower compared to ‘uh’ [31].

Understanding the nature of language function decline is crucial in developing cognitive interventions aimed at improving the quality of life for patients with MCI and dementia [32].

### 2.4. Contribution of This Paper

In this study, cognitive function tests using the Mini-Mental State Examination (MMSE), daily conversations with humans, and daily conversations mediated by AI were conducted for 34 elderly participants. The analysis of the obtained conversation audio data was performed. Previous studies have not sufficiently compared the impact of dialogues conducted by AI agents and human questioners on the speech content of the elderly. Additionally, there is a lack of prior research on how cognitive function levels and dialogue partners influence the frequency of proper nouns and fillers. Diederich et al. (2022) reviewed multiple studies on conversational agents, emphasizing the growing attention to conversational agents in both academia and practise due to advancements in natural language processing. The authors emphasized the significance of tailoring conversational agents to meet specific objectives, while also taking into account the ways users interact with them. Furthermore, they highlighted the need to address the challenges of accommodating a wide range of user groups, including people with disabilities and the elderly, as an important area for future development [33].

While the utilization of AI agents and robots is expected to reduce costs for healthcare professionals and caregivers conducting assessments, differences in the impact on the speech content of patients between human examiners and AI have not been confirmed. This study aims to gain new insights into the nature of communication between humans and AI by targeting a diverse group of elderly users and comparing interactions with both humans and AI.

Additionally, by conducting dialogues with AI and humans and examining the occurrence of fillers, this study may provide important implications for designing AI dialogue systems tailored to the cognitive functions of the elderly. Furthermore, this study offers a new perspective on the issues faced by many traditional cognitive function tests used for diagnosing cognitive impairments, such as testing time, invasiveness, psychological burden on patients, and the cost of necessary equipment and facilities.

## 3. Methods

### 3.1. Experiment Design and Participants

In this study, each participant underwent three tests: a cognitive function test using the MMSE (Mini-Mental State Examination), daily conversations with humans, and daily conversations mediated by AI. The experiments were conducted at a day service center and a Silver Talent Center in Tokyo, with a total of 34 participants. The questioners included both humans and AI. The participants included individuals ranging from those with normal cognitive function to those with dementia. This allowed for the comparison of the impact of different dialogue partners and cognitive functions on the occurrence of fillers and proper nouns in conversations.

According to the World Health Organization (WHO), individuals who are 65 years of age or older are classified as elderly. In Japan, the Ministry of Health, Labor, and Welfare aligns its definition with the WHO, while the Statistics Bureau of Japan also categorizes those aged 65 and above as elderly. Consequently, this study adopts the same classification, considering individuals aged 65 and older as elderly.

The participants consisted of 34 community-dwelling elderly individuals (12 males, 22 females) who were not residing in facilities. Among the 34 participants, 17 were users of day services, and 17 belonged to the silver talent centre in the same area. The silver talent centre is a resource centre for individuals aged 65 and older who have retired from work but still have a desire to work, providing temporary job opportunities through mediation. The day service centre is a facility that provides day services specifically for individuals with dementia. It targets elderly individuals whose cognitive functions have declined but who do not require long-term facility admission, aiming to prevent further cognitive decline and provide opportunities for communication with others.

Additionally, to avoid the influence of dialects, participants residing in Tokyo were targeted. The study was limited to individuals without conditions affecting general conversation hearing or speech, such as hearing loss. Initially, each group included 19 participants, but 2 participants from each group could not be followed up on due to death or transfer during the study period. Participants who agreed to the study’s purpose were included. For elderly participants with cognitive decline, the research content was explained to their families, and consent was obtained. This study was conducted with the approval of the ethics review boards of the University of Tokyo and the Tokyo Metropolitan Institute of Gerontology.

### 3.2. Data Collection

The measurement of cognitive function used the MMSE (Mini-Mental State Examination) [34]. The MMSE is a 30-point cognitive function test composed of 11 items measuring orientation, memory, calculation, language ability, and visuospatial ability, and it can be administered in 6–10 min. Scores of 23 or below are considered indicative of dementia, and scores of 27 or below are suspected of mild cognitive impairment (MCI). In this study, we used the Japanese version translated by Sugimoto, the MMSE-J [35].

For the hearing items in daily conversation, we adopted general hearing items developed in the study by Igarashi et al. (2023) for estimating cognitive function through daily conversation. This question list was created following a strict protocol [36]. These items were created with the cooperation of the University of Tokyo Hospital, based on participant observation of intake interviews conducted by psychologists in the Department of Geriatrics. After organizing the individual questions obtained through participant observation, they were reviewed and revised by five licenced psychologists, and then validated by two researchers. The content designed based on the intake interviews conducted by hospital psychologists covers areas such as family history, physical condition, hobbies, daily routines, memory, time orientation, and spatial orientation. These items were further refined and categorized into six categories: time orientation, spatial orientation, family history, daily routines, physical condition, and interests, resulting in a total of 30 questions (Table 1). Additionally, the target group at the time of creating the question list is similar to the target group in this study, consisting of older adults (a) aged 65 and above, (b) living in the community (not residing in facilities or hospitals), (c) capable of personally expressing consent to participate in the study, and (d) without any diseases or disabilities that affect conversation. From the above, it was considered that these questionnaire items were suitable for the experiment.

This questionnaire is used in both human-to-participant and AI-to-participant conversations. The purpose is to perform a qualitative analysis of the same question to see if there is a difference in responses between the human and AI interviewer. However, if the same question is asked twice in one day, the later question may be influenced by the earlier one (e.g., I answered that question just now). For this reason, the conversations with the human and the AI were conducted on separate days, at least one month apart.

### 3.3. Display Environment of the AI Agent

**Figure 1:**
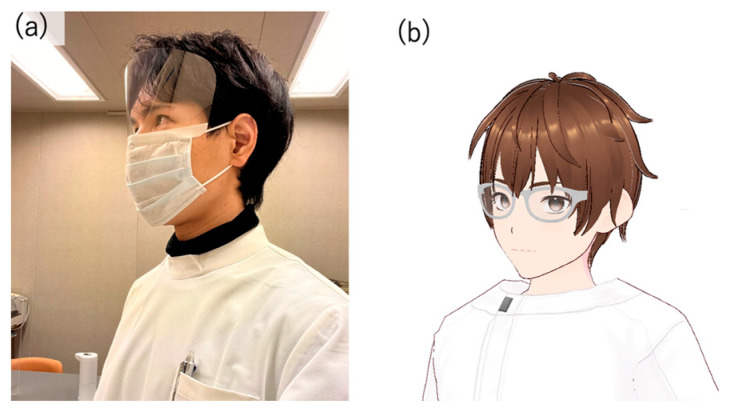
(**a**) the appearance of the author who conducted the interpersonal conversation and (**b**) the appearance of the AI agent modelled based on the author’s appearance.

The implementation of the virtual agent, an avatar with dialogue capabilities provided by AI, utilized several modules. First, since the author conducted the conversations with humans, the AI avatar was also designed to resemble the author in appearance (Figure 1). Figure 1a shows the appearance of the author who conducted the interpersonal conversation, and Figure 1b shows the appearance of the AI agent modelled based on the author’s appearance. VRoid Studio [37] was used for modelling to design an AI agent that resembles the author. Using an avatar whose appearance significantly differs from the person the participants expect to be speaking might influence the test results. Therefore, the goal was to minimize the impact of appearance impressions, acknowledging individual differences.

To display the designed model in the browser, @pixiv/three-vrm [38] was used. @pixiv/three-vrm is an open-source library provided by Pixiv, which loads and displays humanoid 3D avatar models in VRM format using three.js. VRM is based on a 3D standard format that consolidates texture data, materials, and bones into a single file. It is a standard format that includes extensions for handling humanoid models and comes with certain constraints. By running it on a VRM-compatible application, the 3D model can be operated across any compatible app. The AI agent was displayed on a MacBook Pro screen. Additionally, the background of the character included the room of the facility where the dialogue took place, creating an environment where users felt they were conversing with an actual person.

For daily conversations with the AI agent, the Koeiro API [39] was used to generate voice readings. We designed the AI agent’s mouth to move in sync with the voice using a lip-sync function. For recognizing the user’s responses during the conversation, the Web Speech API (Speech Recognition) [40] was used. To avoid difficulties in speech recognition when the agent and participants speak simultaneously, participants wore a lapel microphone, and a system was implemented to recognize voice only while a button was pressed.

### 3.4. Protocols in Conversation Design

**Figure 2:**
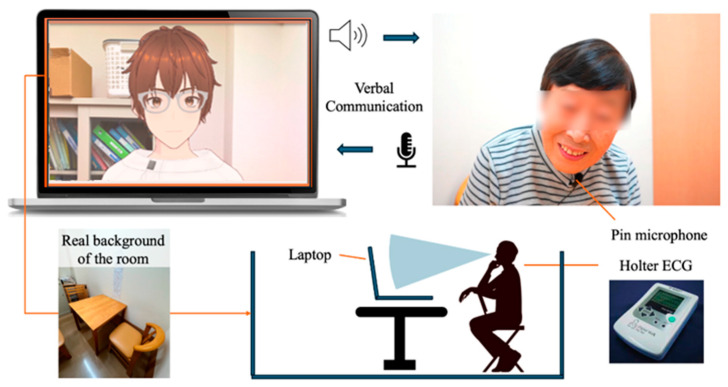
Protocol of daily conversation system for cognitive function estimation by AI agents.

**Figure 3:**
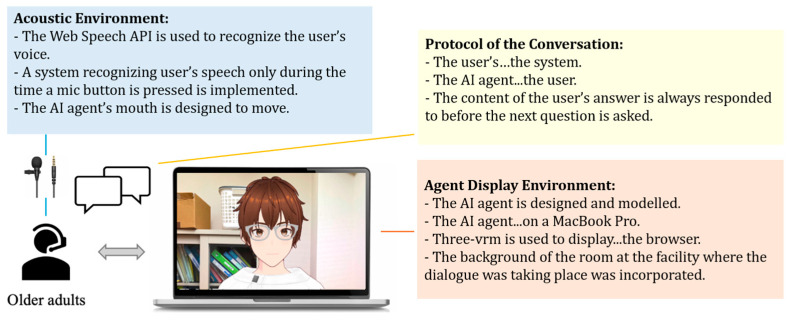
Configuration diagram showing the entire system.

The protocol for conversation design was created using a semi-structured interview method with fixed question content. In a semi-structured interview, the examiner asks questions while changing responses according to the user’s answers. If there is no response to the user’s answer, there is a risk that the user may reduce the quality of their answers or withdraw from the conversation. Therefore, the conversation protocol was designed so that the AI agent always responds to the user’s answer before moving on to the next question. Only the response part corresponding to the user’s answer was implemented using the ChatGPT API [41] (Figure 2). The user sits in front of a PC displaying the AI agent and wears a lapel microphone for voice recognition. Additionally, the user is designed to start the conversation while wearing a Holter ECG monitor (Figure 2 and Figure 3).

### 3.5. Binary Classification of Cognitive Function Using Text Data

The content of conversations in both the AI and human interaction experiments was recorded. This allowed for the qualitative evaluation of the quality of conversations and the reactions of the elderly. The speech content was transcribed by a professional agency. The overall average MMSE score of the participants was 13.47, but the median was 25.5. Therefore, a model was constructed to classify the text data into high cognitive function groups (MMSE score of 26 or higher) and low cognitive function groups.

Generalized language models pre-trained on large corpora have demonstrated excellent performance in natural language tasks. In this study, we used the Japanese pre-trained Bidirectional Encoder Representations from Transformers (BERT), considering the ubiquitous baseline for NLP experiments. BERT provides strong encoding for sentences and text based on Transformers [42]. For the Japanese dictionary, NEologd was used, and MeCab was used for sentence tokenization [43].

Regarding machine learning parameters, we adopted a batch size of 1, a learning rate of 2 × 10^−5^, and an epoch count of 4, based on the study by Igarashi et al. [36]. In this study, to prevent overfitting, which would optimize the model only for the training data and lack generalization, an early stopping program was applied. Learning automatically stopped when it was judged to have been sufficiently trained.

Additionally, the number of subwords (morphemes) that the Japanese pre-trained model used in this study can handle is 512. Since some sentences in the dataset exceeded 512 subwords, any text beyond that was truncated. However, since the average speech for one minute in Japanese is about 300 words and participants were instructed to respond to each question for about one minute, it was considered that sufficient content for classification was included even with truncation. Verification was conducted to determine whether the text data obtained from speech could classify elderly individuals into healthy and cognitive decline groups based on linguistic features when the examiner was a human or an AI agent. Ten-fold cross-validation was performed to ensure that all data were used for both learning and verification.

### 3.6. Analysis of Conversation Quality Using Text Data

The recorded speech content of the participants was analyzed to obtain (a) the number of fillers, (b) the frequency of proper nouns, and (c) the number of instances of listening back. While the question content was common to all participants, the responses varied for each participant, leading to potential differences in the amount of spoken text. For (a) the number of fillers, (b) the frequency of proper nouns, and (c) the number of instances of listening back, the frequency increases with the amount of text. Therefore, it was necessary to analyze these items as ratios to evaluate the quality of the conversation. To this end, a program was created using Google Collaboratory [44] to tokenize the transcribed text data and count the number of fillers and total part-of-speech (POS) tags. The counted fillers were based on the words summarized in previous research on fillers, as follows [21,45] (Table 2).

## 4. Results

The participants of this study included a total of 34 individuals (12 males, 22 females). Participants from the silver talent centre included nine males and eight females, with an average age of 74.35 years and an average MMSE score of 28.70. Participants from the day service centre included three males and fourteen females, with an average age of 83.06 years and an average MMSE score of 13.47. It can be inferred that participants from the silver talent centre performed better functionally on the MMSE test, while participants from the day service centre scored lower on the MMSE test. However, the participation requirements for each facility do not include MMSE test scores, and it is necessary to consider, for example, that some individuals in the silver talent centre may have experienced significant cognitive decline. Therefore, to accurately capture the facts and reflect them in the experiment, the MMSE was conducted in this study. Among the participants from the silver talent centre, the highest MMSE score was 19 and the lowest was 7, while the highest score among participants from the day service centre was 30 and the lowest was 26. Since an MMSE score below 23 is considered the cutoff for suspected dementia, participants with scores above 23 were classified as the high cognitive function group, and those with scores below 23 were classified as the low cognitive function group. The obtained quantitative data were analyzed using Microsoft^®^ Excel^®^ 2021 MSO.

Binary Classification:

When the examiner was a human, the binary classification of the elderly into healthy and cognitive decline groups using text data showed an accuracy of 0.947 for the training data and 0.882 for the test data. When the examiner was an AI agent, the binary classification using text data showed an accuracy of 1.000 for the training data and 0.971 for the test data.

Number of Fillers:

For the number of fillers, the frequency of proper nouns, and the number of instances of listening back, the total POS count was calculated after tokenizing the text, and the ratio was derived using the total POS count as the denominator. For participants from the silver talent centre, the overall average number of fillers was 9.36 words (SD = 3.38). The average number of fillers in conversations with AI was 7.03 words (SD = 2.69), and in conversations with humans, it was 11.69 words (SD = 2.18). For participants from the day service centre, the overall average number of fillers was 11.13 words (SD = 3.03), the average number of fillers in conversations with AI was 11.26 words (SD = 3.30), and in conversations with humans, it was 11.01 words (SD = 2.72).

Frequency of Proper Nouns:

A proper noun refers to a noun that identifies specific entities such as personal names, place names, organization names, and brand names. This distinguishes it from common nouns, which refer to general objects or concepts. In English, proper nouns are typically characterized by beginning with a capital letter in sentences [46]. For participants from the silver talent centre, the average frequency of proper nouns was 1.94 times (SD = 0.83). The average frequency of proper nouns in conversations with AI was 2.55 times (SD = 0.73), and in conversations with humans, it was 1.34 times (SD = 0.31). For participants from the day service centre, the average frequency of proper nouns was 1.37 times (SD = 1.04), the average frequency of proper nouns in conversations with AI was 1.36 times (SD = 1.18), and in conversations with humans, it was 1.39 times (SD = 0.87).

Number of Instances of Listening back:

For participants from the silver talent centre, the average number of instances of listening back was 0.04 times (SD = 0.08). The average number of instances of listening back in conversations with AI was 0.03 times (SD = 0.08), and in conversations with humans, it was 0.04 times (SD = 0.08) (Table 3). For participants from the day service centre, the average number of instances of listening back was 0.27 times (SD = 0.28), the average number of instances of listening back in conversations with AI was 0.37 times (SD = 0.28), and in conversations with humans, it was 0.17 times (SD = 0.25) (Table 4).

## 5. Analysis

Since there was variation in the number of utterances by participants, the number of fillers, the frequency of proper nouns, and the number of instances of listening back were calculated by tokenizing the text and counting the total number of POS tags. The ratios were derived using the total POS count as the denominator for comparison. Tokenization and the calculation of total POS count and fillers were performed using code created in Collaboratory [44] from the transcribed text data. For listening back, the first 10 characters of each section of the participant’s speech were extracted, since listening back typically occurs at the beginning of conversation sections. The number of proper nouns was calculated using morphological analysis.

### 5.1. Comparison of Average Frequency of Fillers by Dialogue Partner and Cognitive Function

First, conversations with humans were compared to conversations with AI without grouping by cognitive function score. 

**Figure 4:**
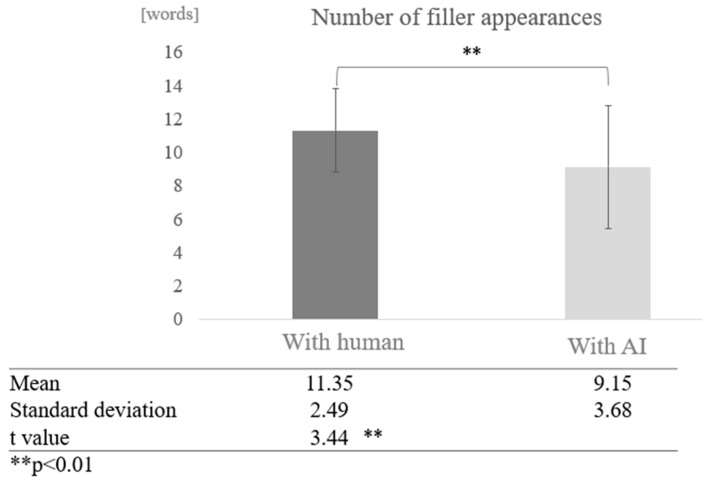
Comparison of the average frequency of fillers based on dialogue partner.

We compared the frequency of fillers in conversations with humans and with AI across all participants. The average number of fillers in conversations with humans was 11.35 words, while the average number of fillers in conversations with AI was 9.15 words, indicating that fillers tended to occur more frequently in conversations with humans. A test of means with a paired sample (two-tailed *t*-test) showed a significant difference (t = 3.44, *p* < 0.01) (Figure 4).

**Figure 5:**
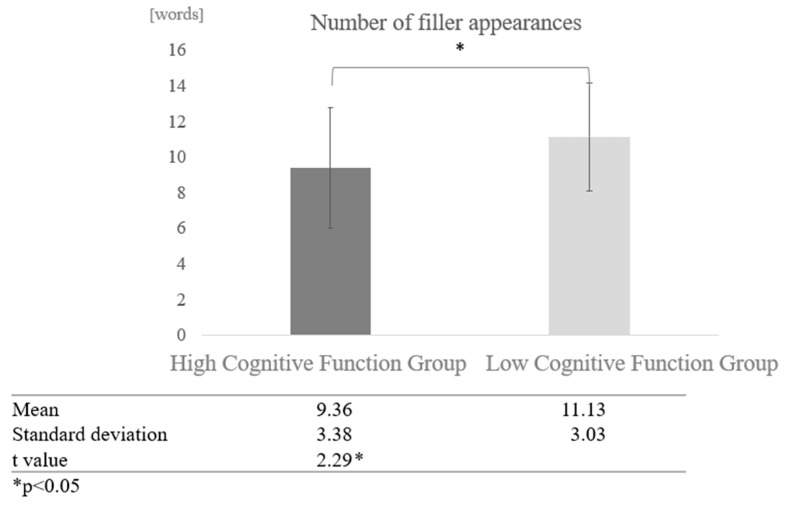
Comparison of the frequency of fillers based on cognitive function levels.

Comparison by cognitive function: We also compared the effect of cognitive function on the degree of filler frequency. The average number of fillers for the entire high cognitive function group, including both human-to-human and human-to-AI conversations, was 9.36 words, while the average number of fillers for the entire low cognitive function group was 11.13 words, indicating that fillers tended to occur more frequently in the low cognitive function group. A two-sample test, assuming that the variances were not equal, revealed a significant difference (t = 2.29, *p* < 0.05) (Figure 5).

**Figure 6:**
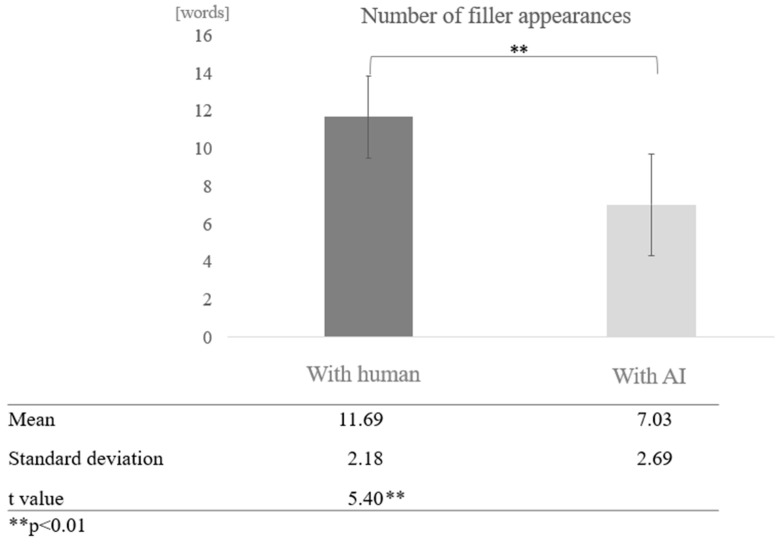
Comparison of the average frequency of fillers based on dialogue partner in the high cognitive function group.

Comparison by dialogue partner for each cognitive function: Next, we compared the average number of filler occurrences by dialogue partner (human/AI agent) between the high and low cognitive function groups. In the high cognitive function group, more fillers tended to be found in conversations with humans than with AI agents, and a test of means with a paired sample (two-tailed *t*-test) revealed a significant difference (t = 5.40, *p* < 0.01) (Figure 6). 

**Figure 7:**
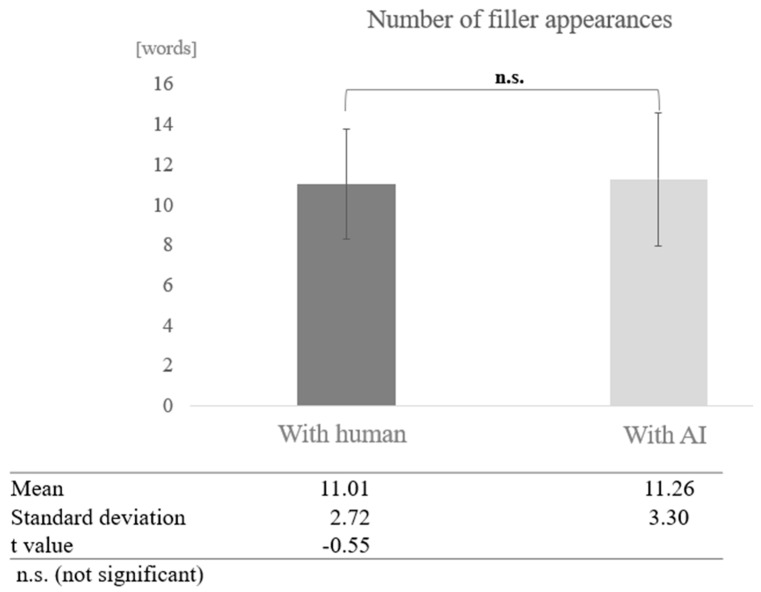
Comparison of the average frequency of fillers based on dialogue partner in the low cognitive function group.

In the low cognitive function group, a test of means with a paired sample (two-tailed *t*-test) revealed no significant difference (t = −0.55, *p* < 0.05) (Figure 7).

**Figure 8:**
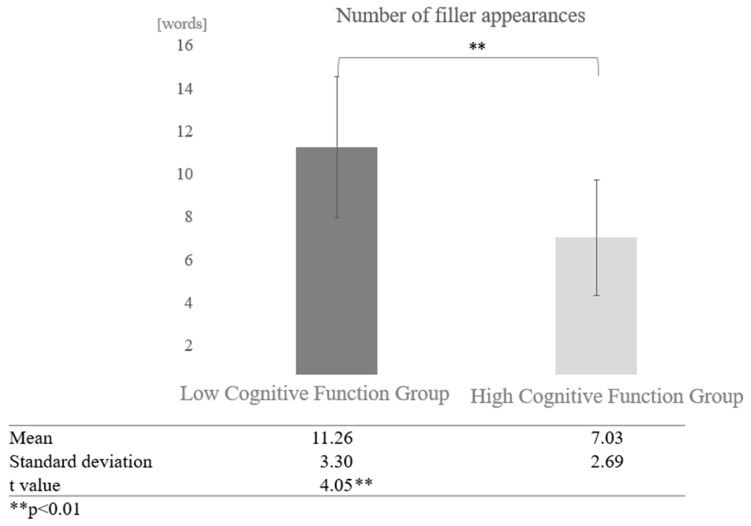
Comparison of the average frequency of fillers based on cognitive function levels in conversations with AI.

Next, a comparison was made regarding the difference between dialogue partners. In AI conversations, a comparison of the number of fillers between the high and low cognitive function groups showed that the low cognitive function group used more fillers. A paired-sample *t*-test (two-tailed) revealed a significant difference (t = 4.05, *p* < 0.01) (Figure 8). 

**Figure 9:**
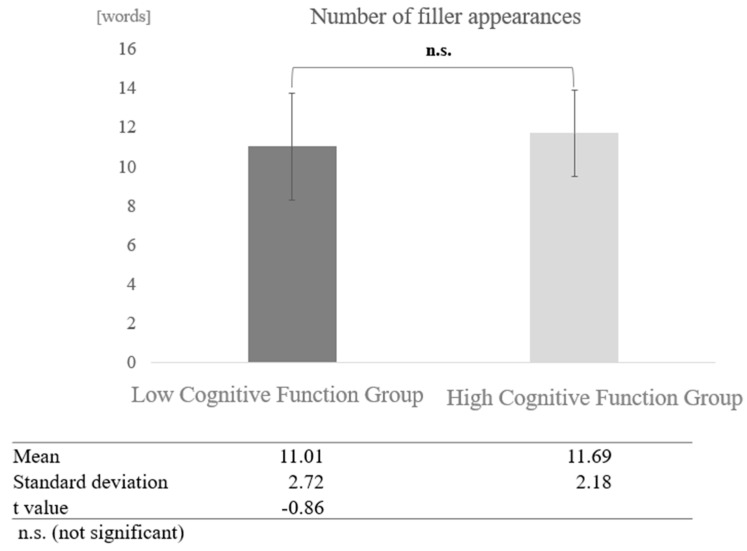
Comparison of the average frequency of fillers based on cognitive function levels in conversations with humans.

In conversations with humans, a paired-sample *t*-test (two-tailed) revealed no significant difference (t = −0.86, *p* < 0.05) (Figure 9).

### 5.2. Comparison of Average Frequency of Proper Nouns by Dialogue Partner

**Figure 10:**
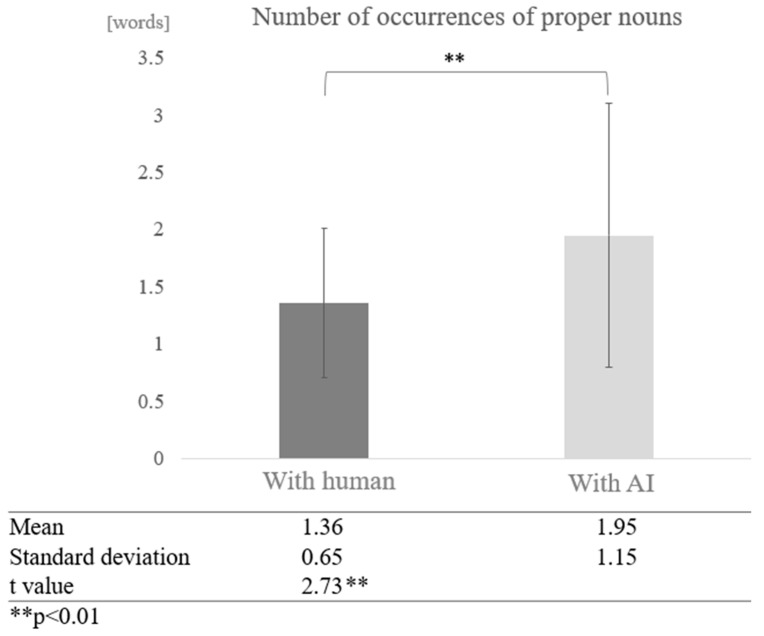
Comparison of the average frequency of proper nouns based on dialogue partner.

We examined whether dialogue partner and cognitive function affect the frequency of proper noun occurrences in conversation. First, participants were not grouped according to their cognitive function scores. The results showed that the average number of occurrences of proper nouns was 1.36 in conversations with humans and 1.95 in conversations with AI, with proper nouns appearing more frequently in conversations with AI. A test of means with a paired sample (two-tailed *t*-test) showed a significant difference between conversations with humans and AI (t = 2.73, *p* < 0.01) (Figure 10).

**Figure 11:**
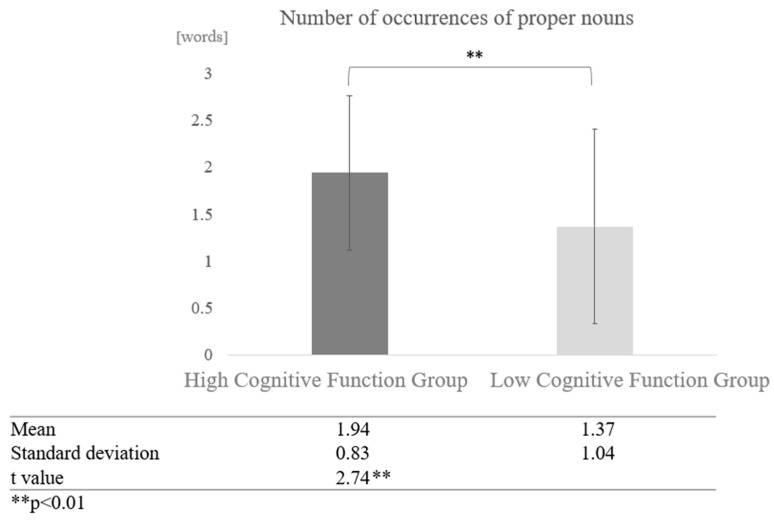
Comparison of the average frequency of proper nouns based on cognitive function levels.

When comparisons were made without grouping by dialogue partner (sum vs. human and vs. AI conversations), the mean number of occurrences of proper nouns was 1.94 for the high cognitive function group as a whole, and 1.37 for the low cognitive function group as a whole. A two-sample test, assuming that the variances were not equal, revealed a significant difference (t = 2.74, *p* < 0.01) (Figure 11).

**Figure 12:**
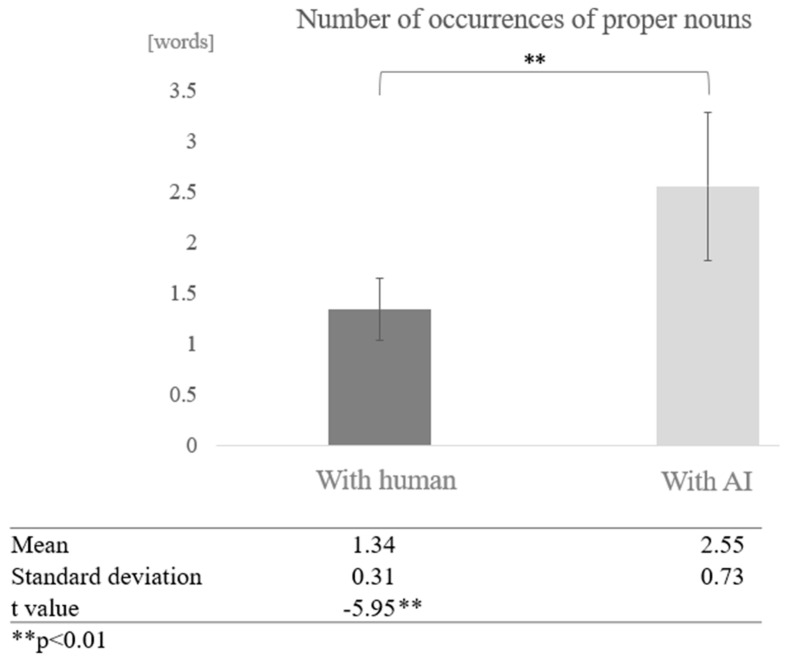
Comparison of the average frequency of proper nouns based on dialogue partner in the high cognitive function group.

Next, we examined the effect of the interaction partner for each cognitive function group. In the high cognitive function group, we compared the means of conversations between humans and AI, and found that the number of proper nouns was higher in conversations between humans and AI. A test of means with a paired sample (two-tailed *t*-test) revealed a significant difference (t = −5.95, *p* < 0.01) (Figure 12).

**Figure 13:**
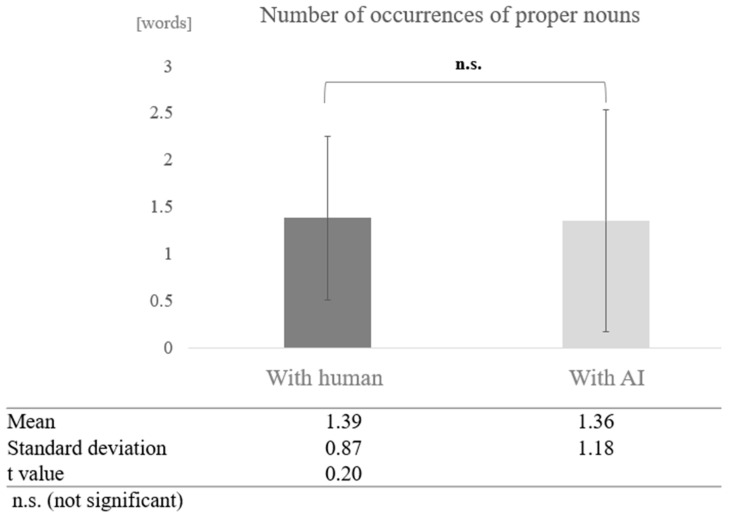
Comparison of the average frequency of proper nouns based on dialogue partner in the low cognitive function group.

In the low cognitive function group, a paired-sample *t*-test (two-tailed) revealed no significant difference (t = 0.20, *p* < 0.05) (Figure 13).

**Figure 14:**
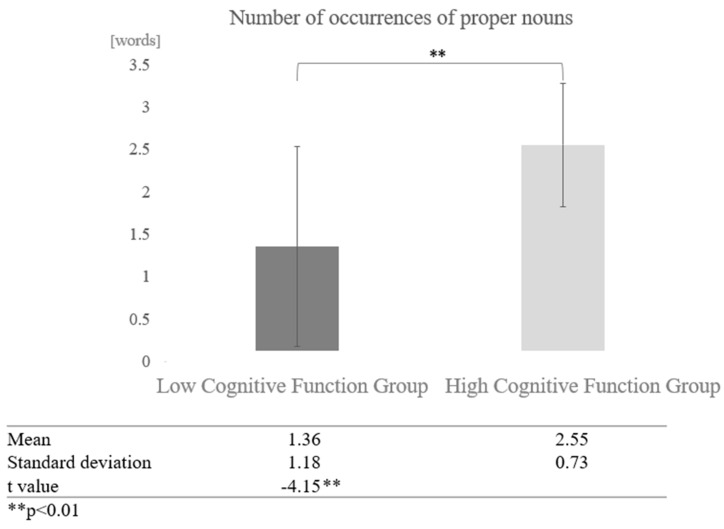
Comparison of the average frequency of proper nouns based on cognitive function levels in conversations with AI.

Next, we examined the effect of cognitive function for each dialogue partner. In conversations with AI, a comparison of the mean values between the high and low cognitive function groups showed that the high cognitive function group used more proper nouns. A paired-sample *t*-test (two-tailed) revealed a significant difference (t = −4.15, *p* < 0.01) (Figure 14).

**Figure 15:**
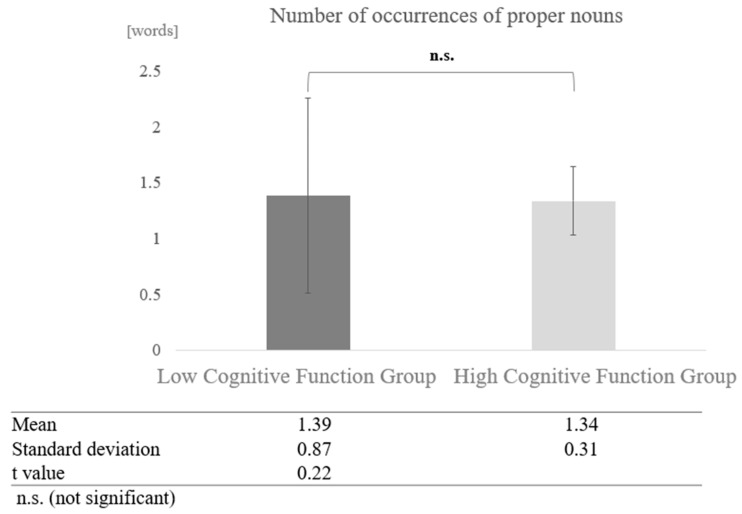
Comparison of the average frequency of proper nouns based on cognitive function levels in conversations with humans.

In contrast, in conversations with humans, a paired-sample *t*-test (two-tailed) revealed no significant difference (t = 0.22, *p* < 0.05) (Figure 15).

### 5.3. Comparison of Average Number of Instances of Listening Back by Dialogue Partner and Cognitive Function

**Figure 16:**
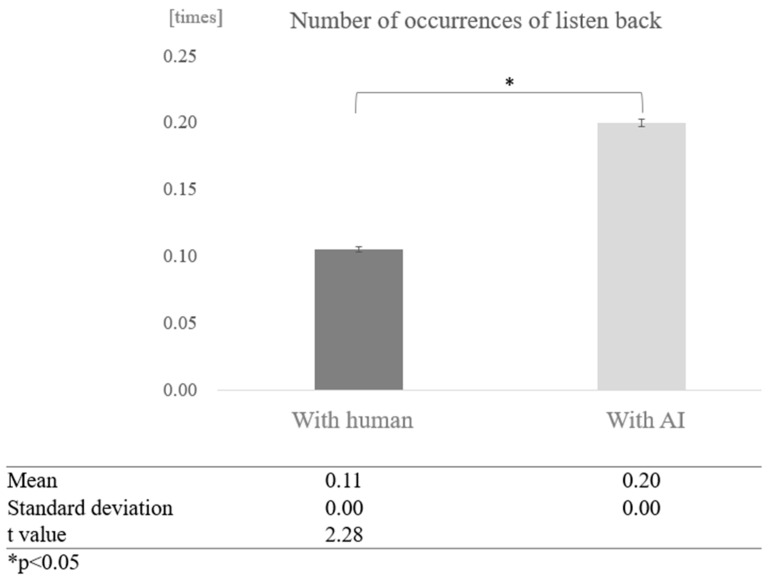
Comparison of the average frequency of listening back based on dialogue partner.

To determine whether dialogue partners and cognitive function affect the number of times a person listens back during a conversation, we first compared conversations with humans and overall conversations with AI, without grouping by cognitive function score. The average number of times of listening back in conversations with humans was 0.11, while the average number of times of listening back in conversations with AI was 0.20, indicating that more listening back was observed in conversations with AI. A test of means with a paired sample (two-tailed *t*-test) showed a significant difference between conversations with humans and AI (t = 2.28, *p* < 0.05) (Figure 16).

**Figure 17:**
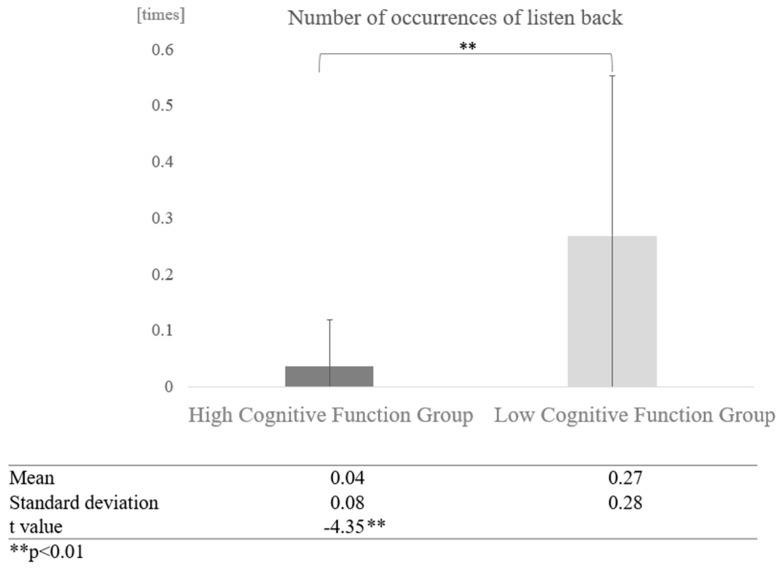
Comparison of the average frequency of listening back based on cognitive function levels.

Furthermore, a comparison of the number of times the high and low cognitive function groups listened back without grouping by interaction partner showed a significant difference (t = −4.35, *p* < 0.01), with the high cognitive function group averaging 0.04 times and the low cognitive function group 0.27 times (Figure 17).

**Figure 18:**
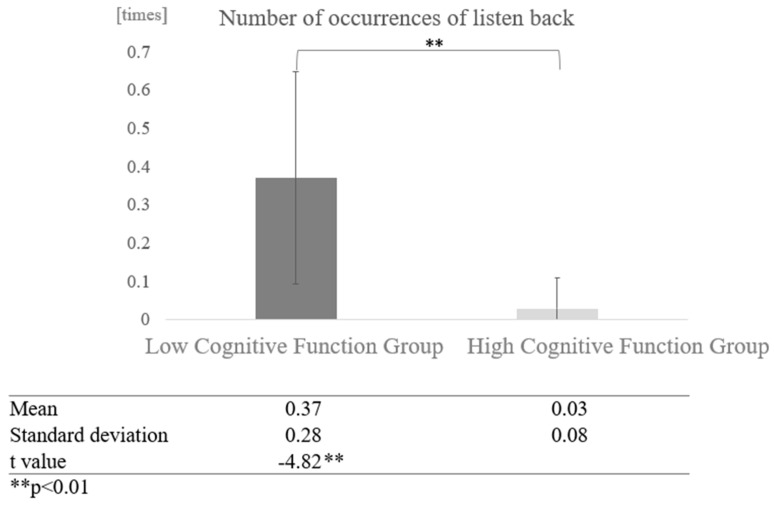
Comparison of the average frequency of listening back based on cognitive function levels in conversations with AI.

Next, a comparison was made for differences in interaction partners: in conversations with the AI, the high cognitive function group and the low cognitive function group listened back more often. A two-sample test, assuming that the variances were not equal, showed that the low cognitive function group listened back significantly more often (t = −4.82, *p* < 0.01) (Figure 18).

**Figure 19:**
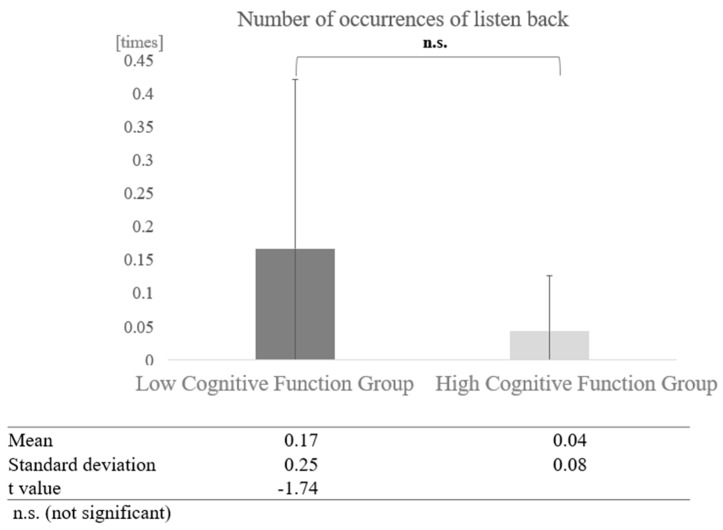
Comparison of the average frequency of listening back based on cognitive function levels in conversations with humans.

In conversations with humans, a paired-sample *t*-test (two-tailed) revealed no significant difference (t = −1.74, *p* < 0.05) (Figure 19).

**Figure 20:**
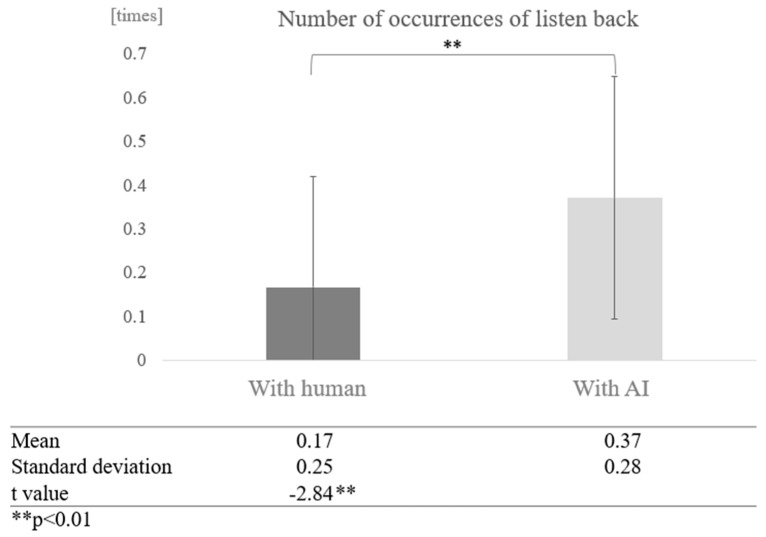
Comparison of the average frequency of listening back based on dialogue partner in the low cognitive function group.

Next, we examined the effect of dialogue partner for each cognitive function group. In the low cognitive function group, a comparison of the mean values between conversations with AI and humans showed that more proper nouns were used in conversations with AI. A paired-sample *t*-test (two-tailed) revealed a significant difference (t = −2.84, *p* < 0.01) (Figure 20).

**Figure 21:**
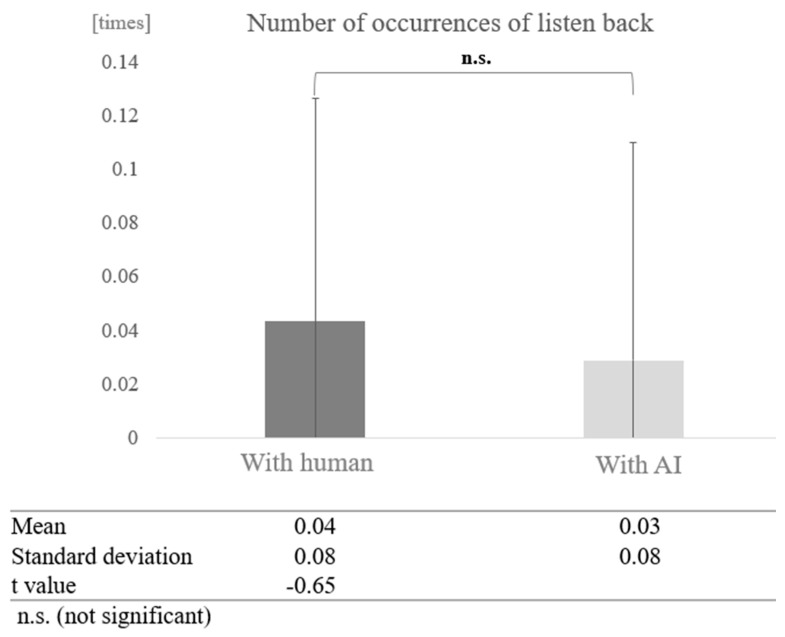
Comparison of the average frequency of listening back based on dialogue partner in the high cognitive function group.

In contrast, in the high cognitive function group, a paired-sample *t*-test (two-tailed) revealed no significant difference (t = −0.65, *p* < 0.05) (Figure 21).

## 6. Discussion

### 6.1. The Impact of Cognitive Function and Dialogue Partners on the Frequency of Fillers

When cognitive function was not considered, it was found that fillers appeared more frequently in conversations with humans than with AI. Furthermore, when comparing averages based on cognitive function without grouping by dialogue partners, it was found that fillers appeared more frequently in the low cognitive function group than in the high cognitive function group. In conversations with AI, the number of fillers was significantly higher in the low cognitive function group. Additionally, regarding the difference in dialogue partners, the number of fillers was higher in conversations with humans in the high cognitive function group.

The reason for the higher frequency of fillers in conversations with humans may be due to the need for thinking time to maintain the flow and rhythm of the conversation. In conversations with AI, there are intervals such as button operations, which may reduce the frequency of fillers. Moreover, Brown (1977) pointed out the “turn-keeping function” of fillers, where producing fillers maintains the speaker’s turn [19]. This “turn-keeping function” of fillers has also been examined from a psychological perspective concerning Japanese fillers [21]. Therefore, in conversations with humans, fillers are used to keep the turn, while in conversations with AI, the dialogue progresses mechanically, eliminating the need for the speaker to secure the turn. For example, when asked, “Where are you from?” one participant answered the AI with “I was born in Busan, Korea, and then moved to Fukui Prefecture”. In contrast, to a human, the same participant said, “Well, actually, I was born in Busan, a corner of Korea, and my father was stationed there, and we moved back”. In the latter response, the participant used fillers like “well” and “uh” to secure the turn and maintain the flow of the conversation. The higher number of fillers in the low cognitive function group during AI conversations suggests that cognitive decline leads to an increase in fillers. Previous studies have shown that the probability of using fillers increases with age [23], consistent with the trends observed in this study.

### 6.2. The Impact of Cognitive Function and Dialogue Partners on the Frequency of Proper Nouns

When cognitive function was not considered, the average frequency of proper nouns was 1.36 times in conversations with humans and 1.95 times in conversations with AI, indicating that proper nouns appeared more frequently in conversations with AI. Furthermore, when comparing averages based on cognitive function without grouping by dialogue partners, it was found that proper nouns appeared more frequently in the high cognitive function group than in the low cognitive function group. In conversations with AI, the number of proper nouns was significantly higher in the high cognitive function group. Additionally, regarding the difference in dialogue partners, the number of proper nouns was higher in conversations with AI in the high cognitive function group.

The result of more proper nouns appearing in the high cognitive function group aligns with existing findings that proper noun production decreases with cognitive decline [47,48].

In conversations with AI, the tendency to use proper nouns more frequently can be attributed to the fact that AI generally requires clear and specific information. While humans often understand vague expressions through context, with AI, users need to use proper nouns to minimize misunderstandings, which may increase their awareness of this need. Additionally, in conversations with humans, people often incorporate episodes or emotions, which can reduce the usage of proper nouns. However, in conversations with AI, the focus may shift towards disclosing facts and exchanging information rather than sharing emotions or stories, which could lead to a higher frequency of proper noun usage. For instance, when asked, ‘What are you passionate about?’ one participant responded to the AI with, ‘Well, I listen to both FM and CDs, and I’ve been listening to classical music all the time’. In this case, proper nouns like ‘FM’ and ‘CD’ were used. In contrast, in a conversation with a human, the same participant said, ‘I’m really into this game I started for dementia prevention right now. It’s Solitaire. I’ve been really into that while listening to classical music’, including an episode but using fewer proper nouns.

### 6.3. The Impact of Cognitive Function and Dialogue Partners on the Frequency of Listening Back

When cognitive function was not considered, more instances of listening back occurred in conversations with AI than with humans. Comparing the high and low cognitive function groups without considering the dialogue partner, the frequency of listening back was significantly higher in the low cognitive function group. Similarly, the frequency of listening back was significantly higher in conversations with AI in the low cognitive function group. Additionally, regarding the difference in dialogue partners, the frequency of listening back was higher in conversations with AI in the low cognitive function group.

The higher frequency of listening back in the low cognitive function group can be attributed to decreased attention and information processing ability due to cognitive decline. It can also be understood in relation to hearing loss. The higher occurrence of listening back in conversations with AI may be because humans can observe the non-verbal aspects of their dialogue partner, aiding understanding. In this study, the human questioner was a nonelderly, certified professional who observed the characteristics and reactions of the participants, possibly reducing the need for clarification. In contrast, AI lacks non-verbal cues and clear intonation, making it harder for participants to understand the speech content. A study found that equipping voice dialogue systems with functions to adjust volume and repeat speech based on the user’s clarification requests (“What?” “Pardon?”) improved intelligibility [49].

### 6.4. Future Work

This study revealed the impact of dialogues by AI agents and human questioners on the speech content of the elderly, but further detailed analysis is needed. Particularly, a deeper understanding of how the questioning methods and dialogue design of AI agents affect the cognitive function evaluation of the elderly is required. Future research should focus on enhancing the dialogue skills of AI agents and customizing them according to the cognitive functions of the elderly.

Additionally, AI agents have the potential to evaluate the cognitive function and Instrumental Activities of Daily Living (IADL) levels of the elderly through dialogues using natural language processing technology. IADL refers to activities necessary for independent living, and assessing them is essential for measuring the daily living independence of the elderly. This method could provide a non-invasive and efficient alternative to traditional face-to-face evaluations, especially in situations where visits are difficult.

Furthermore, based on the data collected by AI agents, personalized care plans for each elderly individual could be designed. The International Classification of Functioning, Disability and Health (ICF) provides a comprehensive framework for evaluating an individual’s health status and related disabilities and social participation levels. It is expected to play an important role in assessing the overall capabilities and disabilities of the elderly in cognitive function evaluations. This approach could improve the quality of life for the elderly who need support for independent living and reduce the burden on caregivers and healthcare professionals.

## 7. Limitations

This study has several limitations. Participants were limited to older people living in the community, and the conversation style was semi-structured interviews. Older people living in nursing homes or hospitals, or older people with different conversational styles, may yield different results. One possible solution to mitigate this limitation would be to conduct future studies in different settings, such as nursing homes or hospitals, and compare results in different settings and conversation styles.

Furthermore, caution must be exercised in generalizing the results due to the limited sample size of participants. In Japan, it is difficult to select older people with cognitive decline as study subjects, and as a result, the sample size for this experiment was small. To conduct this study in Japan with a 95% confidence interval would require approximately 400 participants. However, in Japan, ethical screening for this type of research is strict, and it is not easy to conduct research or experiments on subjects with cognitive decline. Therefore, we believe that this study is significant as a prototype validation to examine the feasibility of such implementation. Future studies may involve working with medical institutions and older people’s care facilities to recruit a wider range of participants, including those with different degrees of cognitive decline.

In addition, the imbalance in the male/female ratio of participants in this experiment was noticeable. This is due to the demographic structure of Japan. In particular, women tend to live longer than men in Japan, which naturally leads to a higher proportion of women in studies targeting older people. Participants were recruited based on the following criteria: they had to be 65 years of age or older, were not institutionalized, lived in Tokyo to avoid dialect effects, and had no hearing or speech impairments. As a result, there was an imbalance between the age and gender of the participants. In order to take into account the age and gender balance as well as personal factors of the participants, future studies should further increase the sample size and validate the results.

Although this study conducted a 30 min dialogue experiment based on routine conversational questions, future research should examine the long-term effects of ongoing dialogue with the AI. A longitudinal study that follows the dialogue over time would provide insight into the sustainability and effectiveness of AI-based communication support systems for older people.

## 8. Conclusions

This study investigated the effects of the dialogue partner (human vs. AI) on conversational characteristics such as frequency of filler, frequency of proper nouns, and instances of listening back, among older participants with different cognitive abilities. The results revealed that there were significant differences in conversational patterns depending on the participants’ cognitive abilities and the type of dialogue partner.

In the filler analysis, the high cognitive function group used an average of 9.36 fillers, 7.03 fillers in AI conversations, and 11.69 fillers in human conversations. The low cognitive function group used an average of 11.13 fillers in AI conversations and 11.01 in human conversations. The high cognitive function group used proper nouns an average of 1.94 times, 2.55 times in AI conversation and 1.34 times in human conversation. The low cognitive function group participants used proper nouns an average of 1.37 times, 1.36 times in AI conversations and 1.39 times in human conversations. Participants in the high cognitive function group averaged 0.04 instances of “listening back,” 0.03 instances in AI conversation, and 0.04 instances in human conversation. 

The results revealed a significant difference between AI and human interactions: significantly more filler was used in AI conversations than in human conversations, and the low cognitive function group used significantly more filler than the participants in the high cognitive function group. In addition, participants with low cognitive function used more filler in conversations with AI, whereas participants with high cognitive function used more filler in conversations with humans. Proper nouns were used significantly more in AI conversations than in human conversations, with participants in the high cognitive function group using more proper nouns than the low cognitive function group. Furthermore, participants with higher cognitive function used more proper nouns in AI conversations.

Finally, with respect to “listening back,” participants asked for significantly more explanations in conversations with the AI than in conversations with humans. Participants in the low cognitive function group asked for explanations more frequently than those in the high cognitive function group, and participants with lower cognitive function asked for explanations more frequently in AI conversations. These results suggest that there are differences in conversation patterns depending on the cognitive function of the participants and whether the conversation partner is a human or an AI.

## Figures and Tables

**Table 1:** Daily conversation used in interpersonal and agent conversations, consisting of 30 questions in 5 areas (copyright Igarashi et al., 2023 [36]).

**(1) Process before coming to the hospital** Q1. Where is your home? Q2. How long did it take you to get here today? Q3. After you left your home, how did you come here? Q4. What time did you leave home to come to the hospital today?
**(2) Life history** Q5. Where were you born? Q6. Do you have any siblings (if so, how many)? Q7. Which elementary school did you attend? Q8. What did you do after elementary school? (Which junior high school did you attend?) Q9. What did you do after graduating junior high school? (Which high school did you attend?) Q10. What do you do for work? (Do you have any memorable stories?) Q11. Are you married? (When was your wedding?) Q12. Do you have any children? (Where do your children live?)
**(3) Normal life** Q13. How do you usually spend your time? (Please tell us your approximate weekly schedule) Q14. What time do you get up in the morning and go to bed? Q15. How often do you go out? (Where do you go most often?) Q16. Do you bathe every day? (Do you bathe in a bathtub?) Q17. How do you prepare your meals? (Do you eat three meals a day?)/What did you eat last night? Q18. How do you clean your house? (How often do you dean your house?) Q19. How do you do your laundry? (How often do you do it?)
**(4) Interests** Q20. What news have you been interested in on TV or the Internet recently? Q21. Please tell me about a sad event that happened to you recently. Q22. Please tell me about a recent unsettling event. Q23. Tell me about a recent event that made you angry. Q24. Tell me ab out a recent event that made you feel bad. Q25. Tell me about a recent event the at surprised you. Q26. Tell me ab out a recent happy event that happened to you. When did it happen? Q27. Tell me ab out someone you admire. Q28. What are you passionate about these days?
**(5) Plans for the rest of the day** Q29. What are your plans for the rest of the day? (How will you get home?) Q30. When was the date of your last visit?

**Table 2:** Classification of Target Fillers.

Type	Category	Word
Vowel Type	A-type	ā, a
	An-type	an, ān
	E-type	ē, e
	En-type	en, ēn
	O-type	o, ō
	N-type	n, $\bar{n}$, u, ū
Lexical Type	Etto-type	etto, ettō, ēto, eto, to
	Ano-type	ano, anō
	Maa-type	ma, mā
Response Words	Hai-type	hai, hāi
	Iya-type	iya, iyā
	Un-type	un, ūn
Others		ano, sono, unto, nanka, nante iuka, mō

**Table 3:** Average Ratio and SD of Obtained Data for Participants from the Silver Human Resources.

			Filler		Listening Back		Proper Nouns	
	Age	MMSE Score	with Human	with AI	with Human	with AI	with Human	with AI
Mean	74.35	28.71	11.69	7.03	0.04	0.03	1.34	2.55
SD	5.3	1.27	2.18	2.69	0.08	0.08	0.31	0.73

**Table 4:** Average Ratio and SD of Obtained Data for Participants from the Day Service Centre.

			Filler		Listening Back		Proper Nouns	
	Age	MMSE Score	with Human	with AI	with Human	with AI	with Human	with AI
Mean	83.06	13.47	11.01	11.26	0.17	0.37	1.39	1.36
SD	4.78	3.84	2.72	3.3	0.25	0.28	0.87	1.18

## Data Availability

Based on the requirements for the ethical review and the protocols outlined by our university for storing and sharing data, our data, which includes information on dementia patients, will be disclosed upon reasonable request.
